# New Low-Melting Triply Charged Homoleptic Cr(III)-Based Ionic Liquids in Comparison to Their Singly Charged Heteroleptic Analogues

**DOI:** 10.3390/ma14102676

**Published:** 2021-05-20

**Authors:** Tim Peppel, Martin Köckerling

**Affiliations:** 1 Leibniz-Institut für Katalyse e.V. (LIKAT), Heterogene Photokatalyse, Albert-Einstein-Str. 29a, 18059 Rostock, Germany; 2 Institut für Chemie, Anorganische Festkörperchemie, Universität Rostock, Albert-Einstein-Str. 3a, 18059 Rostock, Germany; martin.koeckerling@uni-rostock.de; 3 Department Life, Light and Matter, Universität Rostock, 18051 Rostock, Germany

**Keywords:** chromium, ionic liquids, crystal structure, physical properties, melting point

## Abstract

A series of new low-melting triply charged homoleptic Cr(III)-based ionic liquids of the general formula (*R*MIm)_3_[Cr(NCS)_6_] (*R* = methyl, ethyl, *n*-butyl, benzyl) is reported. Their syntheses and properties are described in comparison to their singly charged heteroleptic analogues of the general formula (*R*MIm)[Cr(NCS)_4_*L*_2_] (*R* = methyl, ethyl, *n*-butyl, benzyl; *L* = pyridine, γ-picoline). In total, sixteen new Reineckate related salts with large imidazolium cations are described. Out of these, five compounds were crystallized, and their structures determined by single-crystal X-ray structure analyses. They all consisted of discrete anions and cations with octahedrally coordinated Cr(III) ions. In the structures, various hydrogen contacts interconnect the entities to build up hydrogen bonded networks. Thermal investigations showed relatively low melting points for the homoleptic complexes. The compounds with the [Cr(NCS)_6_]^3−^ anion melt without decomposition and are stable up to 200 K above their melting points. The complex salts with the [Cr(NCS)_4_*L*_2_]^−^ anion, in contrast, start to decompose and lose *L* molecules (Pyr or Pic) already at the melting point.

## 1. Introduction

For more than 30 years, ionic liquids (ILs), designated as organic salts with melting points below 100 °C, have been a broad field of investigation due to their in part unique properties. They exhibit, for instance, large electrochemical windows, liquid ranges, hardly measurable vapor pressures, special solubility characteristics, and applications in catalysis [1,2,3,4,5]. ILs have been often called “designer solvents” and have been used in research fields fitting into the “Green Chemistry” approach. Due to their low vapor pressures at room temperature, they are used as substitutes for common organic solvents. In addition, they exhibit partly exceptional solubility properties for organic, inorganic as well as metal–organic compounds. ILs play an important role as starting materials for N-heterocyclic carbenes (NHCs), which can be applied in a variety of different catalytic reactions [6,7,8].

With the introduction of transition metals into ILs, so-called magnetic ionic liquids (MILs) can be prepared. Currently, these ILs containing metal-based paramagnetic complex ions are a highly investigated subclass because they show magnetic response in addition to the phenomena above-mentioned [9,10,11,12,13]. Furthermore, they have been thoroughly investigated as systems assumed to be magnetic and magnetorheological fluids [14,15].

Solid state investigations of MILs by means of single crystal X-ray diffraction techniques have been applied for 3d, 4d, 5d, and lanthanide metal-containing systems, respectively. For the subclass of 3d metal-based magnetic ILs, most molecular structures are known for Co (e.g., (*R*MIm)_2_[Co*X*_4_], *R* = ethyl, n-butyl; MIm = *N*-methylimidazolium; *X* = Cl, Br) [16,17,18,19,20], Ni (e.g., (*R*MIm)_2_[NiCl_4_], *R* = ethyl, n-butyl) [16,17,18], Cu (e.g., (*R*MIm)_2_[Cu_n_*X*_m_], n = 1, m = 4: *R* = n-butyl, n-dodecyl; *X* = Cl; n = 3, m = 8: *R* = n-butyl; *X* = Cl) [17,21,22], Fe (e.g., (*R*MIm)_2_[FeCl_4_], R = n-butyl) [17], and Zn (e.g., (*R*MIm)_2_[Zn*X*_2_Y_2_], *X* = *Y* = Cl; *R* = n-butyl; *X* = Cl, *Y* = Br; *R* = methyl) [17,23]. The majority of these types of systems contain halide ligands in the complex anions, whereas pseudohalide ligands are scarcely used. The most often used cations are either 1-ethyl-3-methylimidazolium (EMIm) or 1-butyl-3-methylimidazolium (BMIm). Therefore, the melting points of such compounds are far beyond 100 °C.

Over the last decade, we have reported structural information on different series of low-melting imidazolium-based transition MILs. These include 3d metal-based complexes containing Co (e.g., (*R*Im)_2_[CoBr_4_], *R* = 1-butyl-3-methylimidazolium, 1,3-dibutyl-2,4,5-trimethylimidazolium; (*R*MIm)_2_[Co(NC*X*)_4_], *R* = methyl, ethyl, *X* = O, S; and (*R*MIm)[CoBr_3_quin], *R* = ethyl, n-butyl, n-hexyl, n-nonyl, quin = quinoline) [24,25,26,27], Ni (e.g., (*R*Im)_2_[NiBr_4_], *R* = 1,3-dibutyl-2,4,5-trimethylimidazolium; and (*R*MIm)[NiBr_3_*L*], *R* = ethyl, *L* = N-methylimidazole, N-methylbenzimidazole, quinoline, PPh_3_) [27,28], Mn (e.g., polymeric (*R*MIm)[Mn(NCS)_3_], *R* = ethyl; and (DMDIm)[Mn(NCS)_4_], DMDIM = 3,3′-methylenbis(1-methyl-imidazolium)) [29,30], and Cr (e.g., (*R*MIm)[Cr(NCS)_4_*L*_x_], *R* = ethyl, n-butyl, x = 1: *L* = phenanthroline, 2,2′-bipyridine, x = 2: *L* = NH_3_) [31,32,33]. These materials have been synthesized in order to lower the melting point of such substances and to investigate their properties with respect to ionic liquids (ILs).

Salts with high melting points containing Reineckate and analogue anions (Reinecke’s salt, (NH_4_)[Cr(NCS)_4_(NH_3_)_2_]·H_2_O) have been known in the literature for at least 160 years [34,35,36], and have been investigated as chemical actinometers, substances for quantum yield determinations, compounds in charge-transfer photochemistry, and in the analysis of medical products [37,38,39,40]. In particular, complexes exhibiting [Cr(NCS)_4_*L*]^−^ anions [*L* = 1,10-phenanthroline (phen) or 2,2′-bipyridine (bipy)] have been used, for example, in the analytical determination of alkaloids, in the analytical determination of bismuth, and as promising magnetic charge transfer salts [41,42,43].

In this contribution, we extend the series of Reineckate-analogue compounds to triply charged homoleptic as well as singly charged heteroleptic complexes of the general formula (*R*MIm)_x_[Cr(NCS)_4_*L*_2_] (*R* = methyl, ethyl, n-butyl, benzyl; MIm = *N*-methylimidazolium; x = 3: *L* = NCS; x = 1: *L* = Pyr, Pic; Pyr = pyridine; Pic = γ-picoline = 4-methylpyridine). We report on the syntheses, properties, and structures of these three series of imidazolium-based salts. In total, of five substances, crystal and molecular structures were determined by single-crystal X-ray diffraction: (DMIm)_3_[Cr(NCS)_6_], (EMIm)_3_[Cr(NCS)_6_], (BenzMIm)_3_[Cr(NCS)_6_], (BMIm)[Cr(NCS)_4_(Pyr)_2_], and (EMIm)[Cr(NCS)_4_(Pic)_2_], respectively. In addition, all substances were investigated by means of UV/vis and infrared spectroscopy, thermogravimetric analyses (TGA), and differential scanning calorimetry (DSC).

## 2. Materials and Methods

### 2.1. Instrumentation

Elemental analysis for C, H, N, and S was performed on a TruSpec^®®^ Micro device (Leco, St. Joseph, MI, USA). The MIR (MIR = mid-infrared) spectra were recorded by the attenuated total reflectance (ATR) technique on a Bruker Alpha FTIR spectrometer (Bruker Corporation, Billerica, MA, USA) in the region 4000–600 cm^−1^. UV–Vis spectra were recorded on a Lambda 365 device (PerkinElmer, Waltham, MA, USA) in the diffuse reflectance mode for powders or in absorbance mode for solutions. Selective melting points were determined by differential scanning calorimetry (DSC) measurements with a DSC 1 instrument (Mettler-Toledo, Columbus, OH, USA) in the range −60 to 100 °C at a heating rate of 10 K min^−1^ (Ar atmosphere, Al crucible) or STA 449 F3 Jupiter device (Netzsch, Selb, Germany) in the range 25 to 600 °C at a heating rate of 10 K min^−1^ (N_2_ atmosphere, Al crucible). Single-crystal X-ray diffraction measurements were made with an Apex X8 diffractometer (Bruker-Nonius, Billerica, MA, USA) equipped with a CCD detector. The measurements were performed with monochromatic Mo-Kα radiation (λ = 0.71073 Å). The preliminary unit-cell data were obtained from the reflection positions of 36 frames, measured in different directions of reciprocal space. After the completion of the data measurements, the intensities were corrected for Lorentz and polarization effects with the Bruker-Nonius software [44]. Absorption corrections were applied by using the multi-scan method as implemented in SADABS [45].

The structure solutions and refinements were performed with the SHELX program package (vers. 2014) [46]. All non-hydrogen atoms were refined anisotropically. The hydrogen atoms were added at idealized positions and refined in riding models. These data can be obtained free of charge from the Cambridge Crystallographic Data Center: CCDC 1988743 for (DMIm)_3_[Cr(NCS)_6_], CCDC 1988745 for (EMIm)_3_[Cr(NCS)_6_], CCDC 1988741 for (BenzMIm)_3_[Cr(NCS)_6_], CCDC 1988742 for (BMIm)[Cr(NCS)_4_(Pyr)_2_], and CCDC 1988744 for (EMIm)[Cr(NCS)_4_(Pic)_2_].

### 2.2. Materials

All commercially available chemicals were used as received (Sigma-Aldrich, St. Louis, MO, USA, purities > 99%). N-methylimidazole was freshly distilled from KOH in vacuo before use. Ionic liquid precursors were prepared by procedures in the literature [47,48,49,50,51]. K_3_[Cr(NCS)_6_] was synthesized on a multi-gram scale [32,52,53]: A 100 mL aqueous solution of 5.0 g (0.01 mol) chrome alum (chromium(III) potassium sulfate dodecahydrate, KCr(SO_4_)_2_·12H_2_O), and 5.8 g (0.06 mol) KSCN was evaporated to dryness. The residue was extracted exhaustively with ethyl acetate and the resulting solution was evaporated to dryness again. The product K_3_[Cr(NCS)_6_] was obtained as a red-violet solid after final drying at 140 °C in vacuum in high yield as a hygroscopic solid (4.7 g, 90%); elemental analysis for C_6_CrK_3_N_6_S_6_·0.5H_2_O (calcd). C 13.7 (13.7), H 0.2 (0.2), N 16.0 (16.0), S 36.0 (36.5); IR (ν_max_, cm^−1^): 2083 ν(CN), 478 δ(NCS).

### 2.3. Synthesis of (RMIm)_3_[Cr(NCS)_6_]

Samples of the general formula (*R*MIm)_3_[Cr(NCS)_6_] (*R* = methyl, ethyl, n-butyl, benzyl; MIm = methyl-imidazolium) were prepared via direct salt metathesis reaction of 3.0 eq. (*R*MIm)*X* (*X* = Cl, Br, I; 3.0 mmol) and 1.0 eq. K_3_[Cr(NCS)_6_] (0.5 g; 1.0 mmol) in 50 mL acetone with stirring at ambient conditions for 24 h. The precipitate was filtered off and the filtrate solution was evaporated to dryness in vacuum. The residue was dissolved in either dichloromethane or acetone/ethyl acetate (*v:v* = 2:1), filtered, and evaporated to dryness in vacuum again. The final products were recrystallized by dissolution in small amounts of acetone, followed by subsequent precipitation by the slow addition of water. The solids were filtered off, washed thoroughly with water and n-hexane, and finally dried in vacuum. The final products were obtained as red-violet substances in high yields (> 90%).

**(DMIm)_3_[Cr(NCS)_6_]**: Prepared using DMImI [51]. Red-violet solid, yield 75%. M.p. 138 °C, solid-solid transition: 138 °C; elemental analysis for C_21_H_27_CrN_12_S_6_ (calcd.): C 35.2 (36.5), H 4.0 (3.9), N 24.4 (24.3), S 27.8 (27.8); IR (ν_max_, cm^−1^): 2065 ν(CN), 826 ν(CS), 481 δ(NCS); UV/Vis (λ_max_, nm; 23 °C): 413, 552 (powder); 416, 556 (acetone).

**(EMIm)_3_[Cr(NCS)_6_]**: Prepared using commercially available EMImCl. Red-violet solid, yield 91%. M.p. 84 °C; elemental analysis for C_24_H_33_CrN_12_S_6_ (calcd.): C 39.1 (39.3), H 4.5 (4.5), N 22.9 (22.9), S 26.2 (26.2); IR (ν_max_, cm^−1^): 2068 ν(CN), 837 ν(CS), 480 δ(NCS); UV/Vis (λ_max_, nm; 23 °C): 413, 552 (powder); 416, 556 (acetone).

**(BMIm)_3_[Cr(NCS)_6_]**: Prepared using BMImCl [48]. Red-violet solid, yield 95%. M.p. 45 °C; elemental analysis for C_30_H_45_CrN_12_S_6_ (calcd.): C 44.4 (44.0), H 6.5 (5.5), N 20.3 (20.5), S 22.5 (23.5); IR (ν_max_, cm^−1^): 2064 ν(CN), 827 ν(CS), 482 δ(NCS); UV/Vis (λ_max_, nm; 23 °C): 413, 552 (powder); 416, 556 (acetone).

**(BenzMIm)_3_[Cr(NCS)_6_]**: Prepared using BenzMImBr [47,54]. Red-violet solid, yield 97%. M.p. 124 °C; elemental analysis for C_39_H_39_CrN_12_S_6_ (calcd.): C 51.0 (50.9), H 4.2 (4.3), N 18.6 (18.3), S 20.8 (20.9); IR (ν_max_, cm^−1^): 2072 ν(CN), 819 ν(CS), 480 δ(NCS); UV/Vis (λ_max_, nm; 23 °C): 413, 552 (powder); 416, 556 (acetone).

### 2.4. Synthesis of (RMIm)_3_[Cr(NCS)_4_L_2_]

Samples of the general formula (*R*MIm)_3_[Cr(NCS)_4_*L*_2_] (*R* = methyl, ethyl, n-butyl, benzyl; MIm = methyl-imidazolium; *L* = Pyr = pyridine, Pic = γ-picoline = 4-methylpyridine) were prepared via treatment of K_3_[Cr(NCS)_6_] in excessive ligand Pyr or Pic [55,56] to generate substances of the general formula *L*H[Cr(NCS)_4_*L*_2_], followed by the transformation into the corresponding silver salts Ag[Cr(NCS)_4_*L*_2_]. 1.0 eq. (*R*MIm)*X* (*X* = Cl, Br, I; 2.6 mmol) and 1.0 eq. Ag[Cr(NCS)_4_*L*_2_] were stirred at ambient conditions for 24 h in 100 mL of acetone. The resulting suspension was filtered from Ag*X* (*X* = Cl, Br, I), precipitated, and the solvent was removed in vacuum. The residue was dissolved in acetone/ethyl acetate (*v:v* = 2:1), filtered, and evaporated to dryness in vacuum again. The final products were recrystallized by dissolution in small amounts of acetone followed by subsequent precipitation by the slow addition of water. The solid were filtered off, washed thoroughly with water and n-hexane, and finally dried in vacuum. The final products were obtained as red, hygroscopic substances in moderate yields.

**PyrH[Cr(NCS)_4_(Pyr)_2_]**: Dried K_3_[Cr(NCS)_6_] (12.0 g, 23.2 mmol) was suspended in pyridine (11.0 g, 139.0 mmol) and stirred for 4 h at 115 °C in a sealed flask. After cooling to room temperature, the precipitate was washed with portions of acetic acid (10% aqueous solution, 100 mL) and water. Afterward, the precipitate was dissolved in acetone/ethyl acetate (*v:v* = 1:1, 200 mL) and the solution was combined with an aqueous NH_4_Cl solution (20 g in 100 mL H_2_O) and brought to reflux. The resulting solution was evaporated to dryness in vacuum and the precipitate was extracted with acetone. The combined acetonic solutions were evaporated to dryness in vacuum and the resulting red solid was finally dried for several hours in vacuum at 120 °C (11.4 g, 94%). IR (ν_max_, cm^−1^): 2051 ν(CN), 482 δ(NCS).

**Ag[Cr(NCS)_4_(Pyr)_2_]**: PyrH[Cr(NCS)_4_(Pyr)_2_] (5.0 g, 9.6 mmol) was dissolved in 100 mL acetone and slowly added to 200 mL of a vigorously stirred aqueous solution of AgNO_3_ (4.9 g, 28.7 mmol). The resulting precipitate was filtered off, washed excessively with H_2_O, H_2_O/acetone (*v:v* = 1:1) and acetone, and finally dried in vacuum at room temperature for several hours, yielding a light red solid (5.2 g, 98%). IR (ν_max_, cm^−1^): 2078 ν(CN), 472 δ(NCS).

**(DMIm)[Cr(NCS)_4_(Pyr)_2_]**: Prepared from DMImI [51] and Ag[Cr(NCS)_4_(Pyr)_2_]. Red hygroscopic solid, yield 52%. M.p. > 180 °C (dec.); elemental analysis for C_19_H_19_CrN_8_S_4_·H_2_O (calcd.): C 41.0 (40.9), H 3.4 (3.8), N 18.7 (20.1), S 20.5 (23.0); IR (ν_max_, cm^−1^): 2056 ν(CN), 837 ν(CS), 483 δ(NCS); UV/Vis (λ_max_, nm; 23 °C): 535 (powder); 543 (acetone).

**(EMIm)[Cr(NCS)_4_(Pyr)_2_]**: Prepared from commercially available EMImCl and Ag[Cr(NCS)_4_(Pyr)_2_]. Red hygroscopic solid, yield 78%. M.p. 191 °C (dec.); elemental analysis for C_20_H_21_CrN_8_S_4_·H_2_O (calcd.): C 41.6 (42.0), H 4.2 (4.1), N 19.7 (19.6), S 22.8 (22.4); IR (ν_max_, cm^−1^): 2048 ν(CN), 828 ν(CS), 483 δ(NCS); UV/Vis (λ_max_, nm; 23 °C): 535 (powder); 543 (acetone).

**(BMIm)[Cr(NCS)_4_(Pyr)_2_]**: Prepared from BMImCl [48] and Ag[Cr(NCS)_4_(Pyr)_2_]. Red hygroscopic solid, yield 71%. M.p. 207 °C (dec.); elemental analysis for C_22_H_25_CrN_8_S_4_·2H_2_O (calcd.): C 42.7 (42.8), H 4.7 (4.7), N 18.2 (18.1), S 21.0 (20.8); IR (ν_max_, cm^−1^): 2055 ν(CN), 829 ν(CS), 482 δ(NCS); UV/Vis (λ_max_, nm; 23 °C): 535 (powder); 543 (acetone).

**(BenzMIm)[Cr(NCS)_4_(Pyr)_2_]**: Prepared from BenzMImBr [47,54] and Ag[Cr(NCS)_4_(Pyr)_2_]. Red hygroscopic solid, yield 64%. M.p. 185 °C (dec.); elemental analysis for C_25_H_23_CrN_8_S_4_·2.5H_2_O (calcd.): C 45.4 (45.4), H 4.0 (4.3), N 15.8 (17.0), S 20.0 (19.4); IR (ν_max_, cm^−1^): 2043 ν(CN), 820 ν(CS), 482 δ(NCS); UV/Vis (λ_max_, nm; 23 °C): 535 (powder); 543 (acetone).

**PicH[Cr(NCS)_4_(Pic)_2_]**: Dried K_3_[Cr(NCS)_6_] (19.7 g, 38.0 mmol) was suspended in γ-picoline (20.0 g, 214.8 mmol) and stirred for 4 h at 115 °C in a sealed flask. After cooling to room temperature, the precipitate was washed with portions of acetic acid (10% aqueous solution, 100 mL) and water. Afterward, the precipitate was dissolved in acetone/ethyl acetate (*v:v* = 1:1, 300 mL) and the solution was combined with an aqueous NH_4_Cl solution (30 g in 150 mL H_2_O) and brought to reflux. The resulting solution was evaporated to dryness in vacuum and the precipitate was extracted with acetone. The combined acetonic solutions were evaporated to dryness in vacuum and the resulting red solid was finally dried for several hours in vacuum at 130 °C (18.4 g, 86%). Elemental analysis for C_22_H_22_CrN_7_S_4_ (calcd). C 47.0 (46.8), H 4.2 (3.9), N 16.6 (17.4), S 27.9 (22.7). IR (ν_max_, cm^−1^): 2043 ν(CN), 497 δ(NCS).

**Ag[Cr(NCS)_4_(Pic)_2_]**: PicH[Cr(NCS)_4_(Pic)_2_] (5.0 g, 8.8 mmol) was dissolved in 100 mL acetone and slowly added to 100 mL of a vigorously stirred aqueous solution of AgNO_3_ (4.5 g, 26.5 mmol). The resulting precipitate was filtered off, washed with H_2_O, H_2_O/acetone (*v:v* = 1:1) and acetone, and finally dried in vacuum at room temperature for several hours, yielding a reddish solid (4.7 g, 92%). Elemental analysis for C_16_H_14_AgCrN_6_S_4_ (calcd). C 30.2 (33.2), H 2.1 (2.4), N 13.4 (14.5), S 22.2 (22.2). IR (ν_max_, cm^−1^): 2049 ν(CN), 497 δ(NCS).

**(DMIm)[Cr(NCS)_4_(Pic)_2_]**: Prepared from DMImI [51] and Ag[Cr(NCS)_4_(Pic)_2_]. Red solid, yield 80%. M.p. > 220 °C (dec.); elemental analysis for C_21_H_23_CrN_8_S_4_·acetone (calcd.): C 46.3 (46.1), H 4.3 (4.7), N 17.5 (17.9), S 20.6 (20.5); IR (ν_max_, cm^−1^): 2052 ν(CN), 813 ν(CS), 497 δ(NCS); UV/Vis (λ_max_, nm; 23 °C): 537 (powder); 541 (acetone).

**(EMIm)[Cr(NCS)_4_(Pic)_2_]**: Prepared from EMImBr [49] and Ag[Cr(NCS)_4_(Pic)_2_]. Red solid, yield 96%. M.p. 194 °C (dec.); elemental analysis for C_22_H_25_CrN_8_S_4_ (calcd.): C 45.6 (45.4), H 4.1 (4.3), N 16.5 (19.3), S 21.9 (22.1); IR (ν_max_, cm^−1^): 2050 ν(CN), 813 ν(CS), 497 δ(NCS); UV/Vis (λ_max_, nm; 23 °C): 537 (powder); 541 (acetone).

**(BMIm)[Cr(NCS)_4_(Pic)_2_]**: Prepared from BMImCl [48] and Ag[Cr(NCS)_4_(Pic)_2_]. Red solid, yield 76%. M.p. 191 °C (dec.); elemental analysis for C_24_H_29_CrN_8_S_4_·H_2_O (calcd.): C 46.4 (45.9), H 4.7 (5.0), N 16.0 (17.9), S 20.0 (20.4); IR (ν_max_, cm^−1^): 2032 ν(CN), 812 ν(CS), 497 δ(NCS); UV/Vis (λ_max_, nm; 23 °C): 537 (powder); 541 (acetone).

**(BenzMIm)[Cr(NCS)_4_(Pic)_2_]**: Prepared from BenzMImBr [47,54] and Ag[Cr(NCS)_4_(Pic)_2_]. Red solid, yield 61%. M.p. 195 °C (dec.); elemental analysis for C_27_H_27_CrN_8_S_4_·0.5H_2_O (calcd.): C 49.6 (49.7), H 4.5 (4.3), N 15.7 (17.2), S 19.0 (19.6); IR (ν_max_, cm^−1^): 2034 ν(CN), 811 ν(CS), 497 δ(NCS); UV/Vis (λ_max_, nm; 23 °C): 537 (powder); 541 (acetone).

## 3. Results

### 3.1. Syntheses

**Scheme 1:**
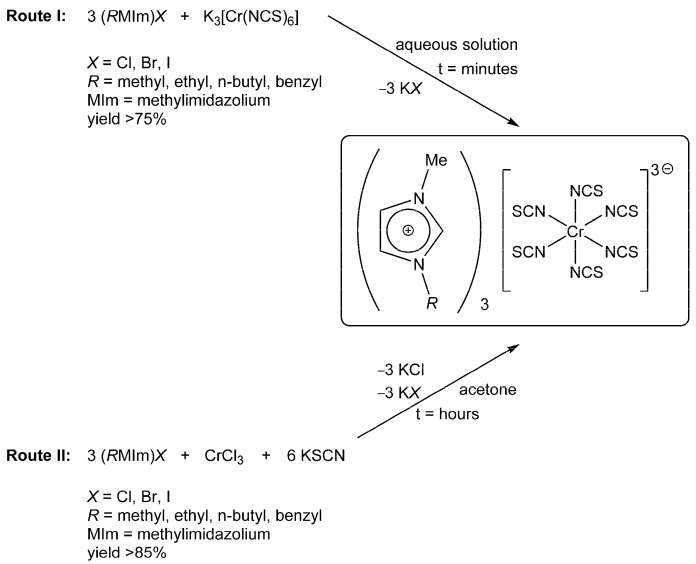
The two general synthetic approaches for the syntheses of complexes of the general formula (*R*MIm)_3_[Cr(NCS)_6_] (*R* = methyl, ethyl, n-butyl, benzyl; MIm = methylimidazolium).

The two different routes to generate homoleptic imidazolium-based Cr^III^ complexes of the general formula (*R*MIm)_3_[Cr(NCS)_6_] (*R* = methyl, ethyl, n-butyl, benzyl) are depicted in Scheme 1. The synthetic approach can be divided into two different routes: (I) the rapid, non-organic solvent, aqueous solution-based salt metathesis of ionic liquid precursors (*R*MIm)*X* (*R* = methyl, ethyl, n-butyl, benzyl; *X* = Cl, Br, I) and K_3_[Cr(NCS)_6_], or (II) the one-pot, direct reaction of ionic liquid precursors (*R*MIm)*X* with CrCl_3_ and KSCN in acetonic solution. Both routes lead to the same low-melting Cr(III)-based ionic liquid (*R*MIm)_3_[Cr(NCS)_6_] materials with high yields. The overall yield of route I is slightly below the overall yield of route II, mainly because of the work-up strategy. Starting in route II with CrCl_3_ and using an organic solvent makes the product separation more effective than in the aqueous strategy used in route I.

**Scheme 2:**
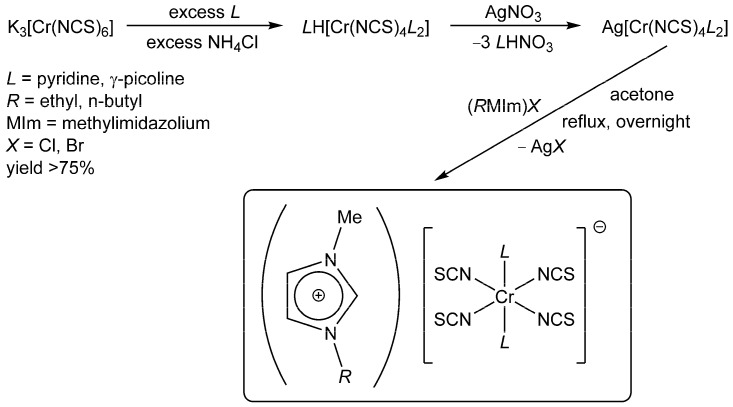
Multi-step synthetic approach to generate complexes with the general formula (*R*MIm)[Cr(NCS)_4_*L*_2_] (*R* = ethyl, n-butyl; MIm = *N*-methylimidazolium; *L* = pyridine, γ-picoline).

The route for the heteroleptic complexes of the formula (*R*MIm)[Cr(NCS)_4_*L*_2_] (*R* = ethyl, n-butyl; MIm = *N*-methylimidazolium; *L* = pyridine, γ-picoline) is given in Scheme 2. The general synthetic procedure includes a three-step reaction pathway starting from K_3_[Cr(NCS)_6_]. In a first step, K_3_[Cr(NCS)_6_] is reacted with and in excess *L* (*L* = pyr, pic), afterward the *L*H[Cr(NCS)_4_*L*_2_] compound is further transferred into its Ag salt. A subsequent salt metathesis reaction with ionic liquid precursors (*R*MIm)*X* leads to the final products (*R*MIm)[Cr(NCS)_4_*L*_2_] in moderate yields.

### 3.2. Physicochemical Properties: Electronic and Infrared Data

Octahedral Cr^III^ complexes exhibit two distinct spin allowed transitions in the visible region, namely ^4^T_2g_ ← ^4^A_2g_ (low energy) and ^4^T_1g_(F) ← ^4^A_2g_ (higher energy). An additional third short-wave band, ^4^T_1g_(P) ← ^4^A_2g_ (highest energy), is usually superimposed by charge-transfer bands [57]. Electronic spectra for complexes bearing the [Cr(NCS)_4_*L*_2_]^−^ (*L* = eg. NH_3_, amines, phosphines) anion are reported in the literature in detail. For N-donor ligands, the spectrochemical series is reported to be NCS^−^ < Pyr < NH_3_ [58]. For Reineckates (*L* = NH_3_), transitions are reported at 392 and 503 nm, and for *L* = Pyr at 545 nm, respectively, in the solid state under diffuse reflectance conditions [58].

**Figure 1:**
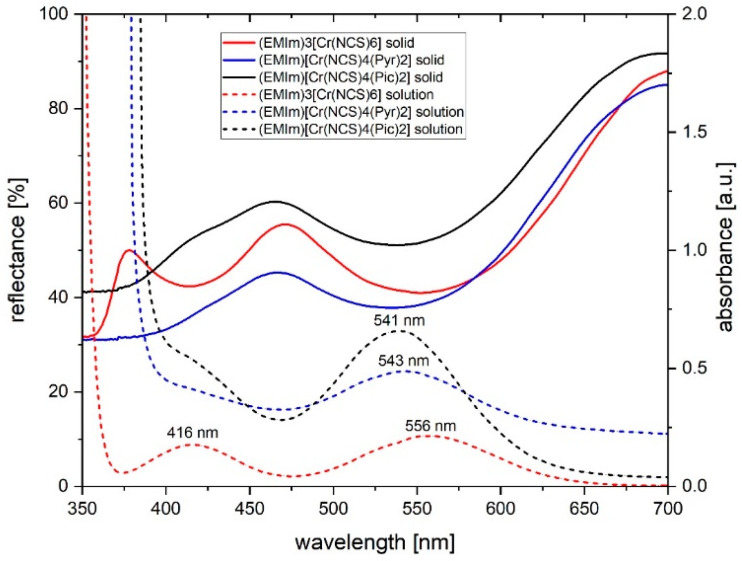
UV/Vis spectra of solid samples (solid lines) and acetonic solutions (dashed lines) of samples of (EMIm)_x_[Cr(NCS)_4_*L*_2_] (x = 3: *L* = NCS; x = 1: *L* = Pyr, Pic).

UV/Vis spectra in diffuse reflectance as well as in an acetonic solution of selected (EMIm)_x_[Cr(NCS)_4_*L*_2_] (x = 3: *L* = NCS; x = 1: *L* = Pyr, Pic) compounds are depicted in Figure 1. Full information on all transitions are given in the Experimental section and maxima for compounds exhibiting the [Cr(NCS)_6_]^-^ anion are found at 413 (416) and 552 (556) nm, for *L* = Pyr at 535 (543) nm, and for *L* = Pic at 537 (541) nm, respectively. These values closely resemble the ones found in the literature and the ligand field strength is also in accordance [31,32,33,56,58].

### 3.3. Thermal Properties

All substances were subjected to thermogravimetric analyses (TGA) and differential scanning calorimetry (DSC) measurements to investigate their thermal as well as melting behavior. The melting points were detected as endothermic peaks in the DSC measurements and are listed in the Experimental section and in Table 1.

Compounds of the formula (*R*MIm)_3_[Cr(NCS)_6_] (*R* = methyl, ethyl, n-butyl, benzyl) can be melted and recrystallized without decomposition, whereas the comparable compounds with pyridine or picoline ligands melt with decomposition. The melting points of the hexaisothiocyanato complexes can be lowered by extending the alkyl chain in the imidazolium-based cation from 138 °C in (DMIm)_3_[Cr(NCS)_6_] to 45 °C in (BMIm)_3_[Cr(NCS)_6_]. This behavior can be found in a variety of other ionic liquids and ionic liquid complexes, but these melting points are exceptionally low for triply charged Cr(III)-based compounds. A recent study has shown that low charge density can be attributed to the low melting points of systems with high charges [59]. Hence, this behavior could be present, particularly in systems also exhibiting the symmetric [Cr(NCS)_6_]^3−^ complex anion, leading to the observed low melting points. In contrast, the melting points were not as low as for the comparable doubly charged complexes (e.g., the low-viscosity isothiocyanato-based compound [EMIm]_2_[Co(NCS)_4_]) [26].

**Figure 2:**
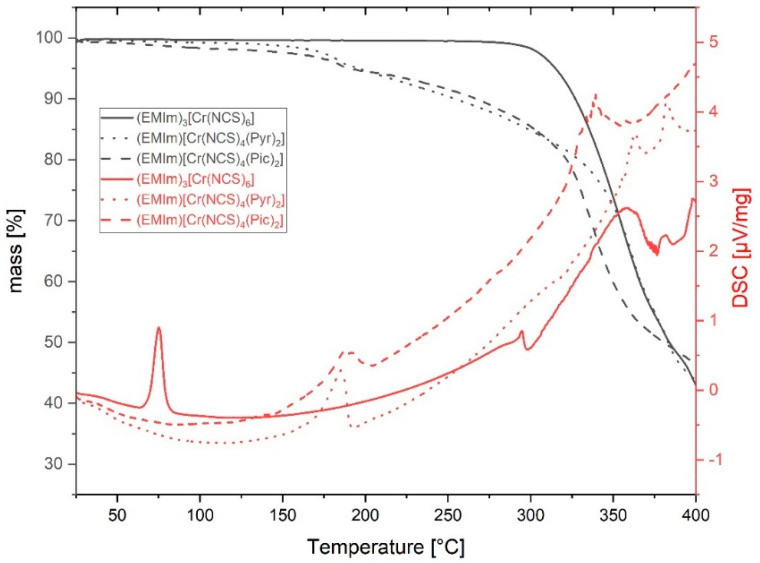
Thermogravimetric (black) and DSC (red) results of samples of (EMIm)_x_[Cr(NCS)_4_*L*_2_] (x = 3: *L* = NCS; x = 1: *L* = Pyr, Pic) in a N_2_ atmosphere.

Decomposition curves of the three selected substances (EMIm)_x_[Cr(NCS)_4_*L*_2_] (x = 3: *L* = NCS; x = 1: *L* = Pyr, Pic) in a nitrogen atmosphere in the temperature range 25 to 400 °C are shown in Figure 2. From Figure 2, it can be seen that the decomposition of (EMIm)_3_[Cr(NCS)_6_] starts at ca. 300 °C and the compounds stay undecomposed in the liquid state of a range of more than 200 K. Compounds (EMIm)[Cr(NCS)_4_*L*_2_] (*L* = Pyr, Pic) melt just below 200 °C by loss of ca. 5% mass. This can be attributed to the beginning release and evaporation of pyridine or picoline ligands into the gas phase. The gradual decomposition is further accelerated above 300 °C, which is comparable to the behavior of (EMIm)_3_[Cr(NCS)_6_] and comparable amine-based isothiocyanate chromate(III) complexes [56].

### 3.4. Crystal Structures

Single crystals suitable for X-ray structure determinations of (*R*MIm)_3_[Cr(NCS)_6_] (*R* = methyl, ethyl, benzyl), (BMIm)[Cr(NCS)_4_(Pyr)_2_], and (EMIm)[Cr(NCS)_4_(Pic)_2_] were obtained by slow evaporation acetonic solutions of the three compounds at ambient pressure and temperatures of 25 °C over a period of one week. Crystallographic data and structure-refinement parameters of all five compounds can be found in Table 2. Additional selected interatomic distances for the complex anions and hydrogen-bond geometries are given in Table 3.

Crystal structure determinations of ammonium-based [Cr(NCS)_6_]^3−^ compounds have so far only been known for the two compounds (NH_4_)_3_[Cr(NCS)_6_]·acetone and (Me_4_N)_3_[Cr(NCS)_6_] [60]. The crystal structure of an additional phosphonium-based compound is known for (Ph_4_P)_3_[Cr(NCS)_6_]·acetonitrile [61]. All these salts exhibit high melting points far beyond 100 °C, hence the crystal structure determinations of the new low-melting salts can provide a high impact to the structural information database.

**Figure 3:**
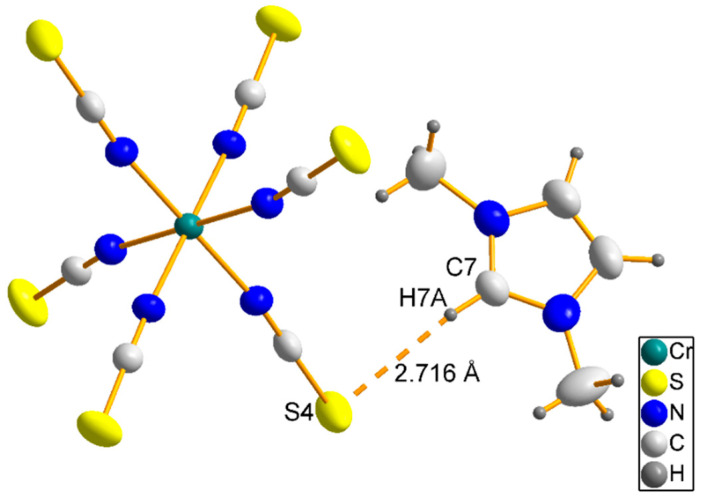
Structure of the cation–anion pair in (DMIm)_3_[Cr(NCS)_6_] with atom labeling scheme depicting the shortest C–H···S contact (thermal ellipsoids are drawn at the 30% probability level).

**Figure 4:**
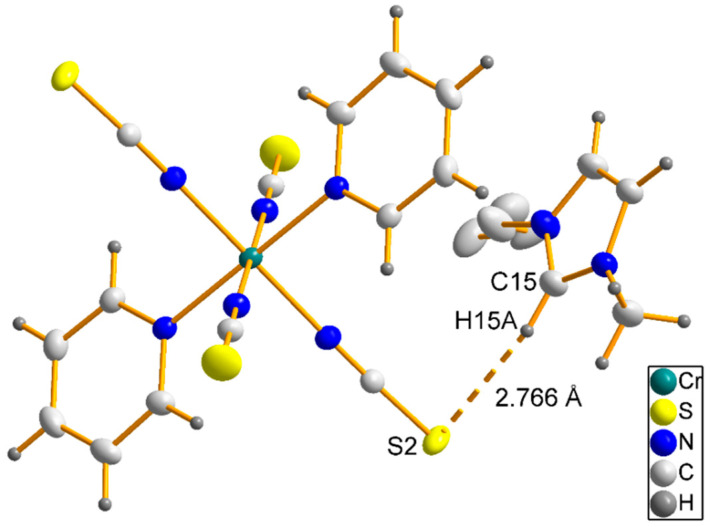
Structure of the cation–anion pair in (BMIm)[Cr(NCS)_4_(Pyr)_2_] with atom labeling scheme depicting the shortest C–H···S contact (thermal ellipsoids are drawn at the 30% probability level; hydrogen atoms at the disordered n-butyl chain omitted for clarity).

**Figure 5:**
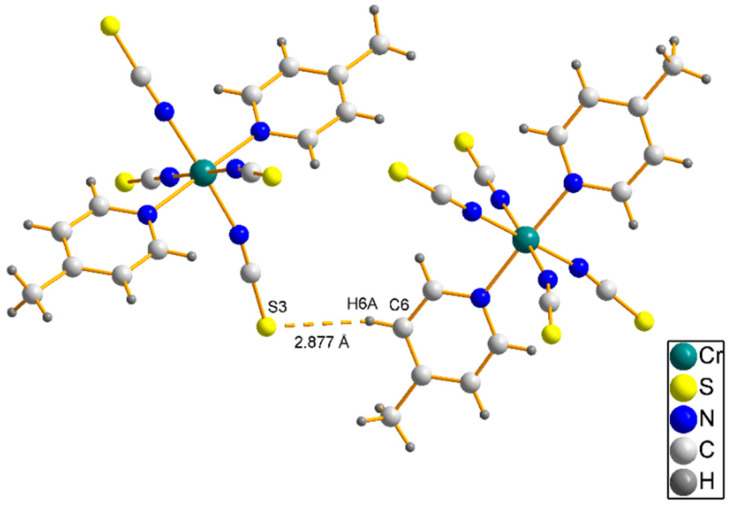
Anion–anion pairing in (EMIm)[Cr(NCS)_4_(Pic)_2_] via short hydrogen contacts with atom labeling scheme depicting the shortest C–H···S contact (thermal ellipsoids are drawn at the 30% probability level; disordered cation omitted for clarity).

**Figure 6:**
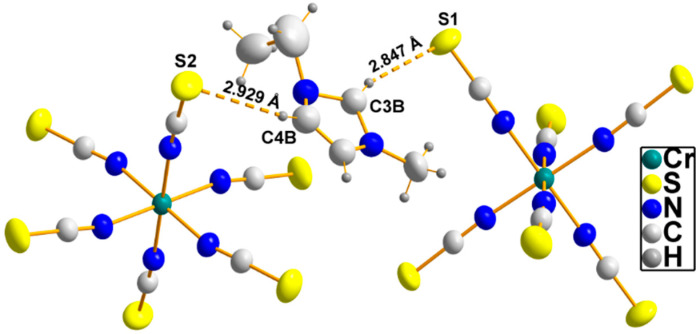
Anion–cation pairing in (EMIm)_3_[Cr(NCS)_6_] via short hydrogen contacts depicting the shortest C–H···S contact (thermal ellipsoids are drawn at the 30% probability level).

**Figure 7:**
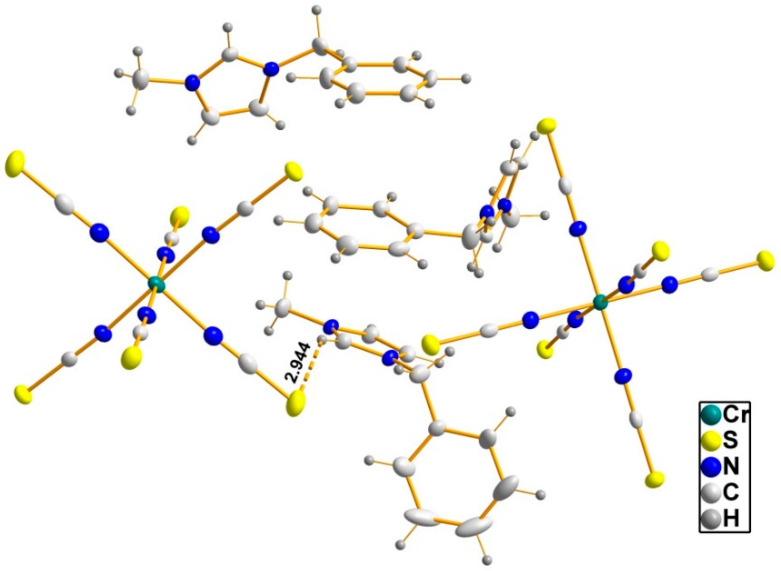
Anion–cation pairing in (BenzMIm)_3_[Cr(NCS)_6_] via short hydrogen contacts depicting the shortest C–H···S contact (thermal ellipsoids are drawn at the 30% probability level).

Thermal ellipsoid plots of the structural units in (DMIm)_3_[Cr(NCS)_6_], (BMIm)[Cr(NCS)_4_(Pyr)_2_], (EMIm)[Cr(NCS)_4_(Pic)_2_], (EMIm)_3_[Cr(NCS)_6_], and (BenzMIm)_3_[Cr(NCS)_6_] are given in Figure 3, Figure 4, Figure 5, Figure 6 and Figure 7, respectively. Figure 3, Figure 4, Figure 6 and Figure 7 also depict the shortest hydrogen contacts (C–H···S) between the isothiocyanato ligands of the complex anions and the hydrogen atoms of the cations. Except for these shortest hydrogen contacts between cations and anions, additional hydrogen anion–anion interactions seem to be present in the molecular structure of (EMIm)[Cr(NCS)_4_(Pic)_2_] (Figure 5). This behavior has not yet been reported in comparable molecular structures of Reineckate-related compounds in the literature.

In all five structurally characterized compounds, the complex anions [Cr(NCS)_6_]^3−^ and [Cr(NCS)_4_(*L*)_2_]^–^ (*L* = Pyr, Pic) consist of Cr^III^ ions coordinated octahedrally by either six isothiocyanato ligands with average Cr–N bond lengths of 2.00–2.01 Å or four isothiocyanato ligands and two additional neutral amine-based ligands. The average Cr–N_amine_ bond length equals 2.08 Å. The bond lengths are in good agreement with those found in other complexes with comparable complex anions [31,32,33,60,61,62,63]. The presence of extended hydrogen-bonding networks further influences the bond angles (see Table 3).

All angles and bond lengths within the methylimidazolium-based cations (*R*MIm)^+^ (*R* = methyl, ethyl, n-butyl, benzyl) are in accordance with values reported in the literature for the corresponding substances (DMIm)[Co(NCO)_4_] [24], (EMIm)[*M*Br_3_*L*] (*M* = Co: *L* = quinoline; *M* = Ni: *L* = quinoline, N-methylbenzimidazole, N-methylimidazole, PPh_3_) [25,28], (EMIm)[Cr(NCS)_4_(NH_3_)_2_] and (BMIm)[Cr(NCS)_4_*L*] (*L* = 1,10-phenanthroline, 2,2′-bipyridine) [31,32,33], or (BenzMIm)[PF_6_] and (BenzMIm)Cl·0.25H_2_O [64,65], respectively.

## 4. Conclusions

The subclass of ionic liquids bearing Reineckate-derived complex anions (Reinecke’s salt, (NH_4_)[Cr(NCS)_4_(NH_3_)_2_]·H_2_O) could be successfully extended to substances of the general formula (*R*MIm)_x_[Cr(NCS)_4_*L*_2_] (*R* = methyl, ethyl, n-butyl, benzyl; x = 3: *L* = NCS; x = 1: *L* = Pyr, Pic). The homoleptic triply charged salts of the general formula (*R*MIm)_3_[Cr(NCS)_6_] exhibit relatively low melting points and can be transferred into the liquid state without decomposition. These model substances could find a broader application in Cr(III)-based catalytic transformations (e.g., olefin polymerizations or tri- and tetramerization reaction, respectively). Comparable (*R*MIm)[Cr(NCS)_4_*L*_2_] complexes have shown that they undergo decomposition by the release of amine ligands upon melting. The melting points are almost 100 K higher than the melting point of the comparable (*R*MIm)_3_[Cr(NCS)_6_] compounds. Thorough single-crystal X-ray investigations revealed that the substances are designated by extended hydrogen contact networks in the solid state, whereas additional anion–anion contacts are present in the respective (*R*MIm)[Cr(NCS)_4_(Pic)_2_] compounds. This is the first example of such behavior to be reported for this subclass of Reineckate-related substances.

## Figures and Tables

**Table 1:** Melting points [°C] derived from the DSC measurements of substances of the general formula (*R*MIm)_3_[Cr(NCS)_6_] and (*R*MIm)[Cr(NCS)_4_*L*_2_] (*R* = Methyl, Ethyl, Benzyl; *L* = Pyr, Pic; dec.: with decomposition).

*R*	(*R*MIm)_3_[Cr(NCS)_6_]	(*R*MIm)[Cr(NCS)_4_(Pyr)_2_]	(*R*MIm)[Cr(NCS)_4_(Pic)_2_]
Methyl	138	180 (dec.)	220 (dec.)
Ethyl	84	191 (dec.)	194 (dec.)
n-Butyl	45	207 (dec.)	191 (dec.)
Benzyl	124	185 (dec.)	195 (dec.)

**Table 2:** Crystallographic data and structure-refinement parameters for (*R*MIm)_3_[Cr(NCS)_6_] (*R* = Methyl, Ethyl, Benzyl), (BMIm)[Cr(NCS)_4_(Pyr)_2_], and (EMIm)[Cr(NCS)_4_(Pic)_2_].

	(DMIm)_3_[Cr(NCS)_6_]	(EMIm)_3_[Cr(NCS)_6_]	(BenzMIm)_3_[Cr(NCS)_6_]	(BMIm)[Cr(NCS)_4_(Pyr)_2_]	(EMIm)[Cr(NCS)_4_(Pic)_2_]
Formula	C_21_H_27_CrN_12_S_6_	C_24_H_33_CrN_12_S_6_	C_39_H_39_CrN_12_S_6_	C_22_H_25_CrN_8_S_4_	C_22_H_25_CrN_8_S_4_
Fw [g mol^−1^]	691.90	733.98	920.18	581.74	581.74
Crystal system	triclinic	trigonal	monoclinic	triclinic	orthorhombic
Space group	*P* $\bar{1}$	*R* $\bar{3}$	*P*2_1_/*c*	*P* $\bar{1}$	*Pbca*
*a* [Å]	9.4116 (6)	18.220 (3)	18.6109 (8)	8.3939 (5)	17.1199 (9)
*b* [Å]	10.0862 (6)	19.198 (4)	13.4405 (6)	9.9145 (6)	15.5043 (8)
*c* [Å]	18.630 (1)	18.220 (3)	17.9848 (8)	17.447 (1)	20.981 (1)
*α* [°]	87.525 (4)	90.0	90.0	101.441 (2)	90.0
*β* [°]	81.527 (3)	90.0	97.967 (2)	91.854 (3)	90.0
*γ* [°]	75.893 (3)	120.0	90.0	97.682 (2)	90.0
*V* [Å^3^], *Z*	1696.4 (2), 2	5519 (2), 6	4455.3 (3), 4	1407.7 (1), 2	5569.0 (6), 8
*T* [K]	293	293	173	173	173
*ρ*_calcd._ [g cm^−3^]	1.355	1.325	1.372	1.372	1.388
*μ* [mm^−1^]	0.73	0.679	0.576	0.724	0.732
Reflections collected	34,948	46,540	37,816	22,262	24,664
Independent reflections	11,424	4056	9172	6917	6939
Parameters	364	179	526	347	373
*GooF*	1.00	1.02	0.96	1.02	0.99
*R*[*F*^2^ > 2*σ*(*F*^2^)]	0.047	0.042	0.056	0.045	0.047
*wR*(*F*^2^) ^(^^*a*^^,^^*b*^^)^	0.153	0.140	0.114	0.122	0.133
Δ*ρ* (max/min), e Å^–3^	0.48/−0.33	0.35/−0.34	0.36/−0.33	0.98/−0.70	0.49/−0.51

(*a*) $R1=\;\frac{\sum^{}\left|\left|F_{0}\right|-\left|F_{c}\right|\right|}{\sum^{}\left|F_{0}\right|}$; wR2 = $\sqrt{\frac{\sum^{}w{\left(F_{o}^{2}-F_{c}^{2}\right)}^{2}}{\sum^{}w{\left(F_{o}^{2}\right)}^{2}}};\left(b\right)\;w=1/[(\sigma^{2}\left(F_{o}^{2}\right)+\left(A*P{)}^{2}+B*P\right];P=\frac{F_{o}^{2}+2F_{c}^{2}}{3}$.

**Table 3:** Selected interatomic distances [Å] for the complex anions and hydrogen-bond geometries in (*R*MIm)_3_[Cr(NCS)_6_] (*R* = Methyl, Ethyl, Benzyl), (BMIm)[Cr(NCS)_4_(Pyr)_2_], and (EMIm)[Cr(NCS)_4_(Pic)_2_].

Bond	(DMIm)_3_[Cr(NCS)_6_]	(EMIm)_3_[Cr(NCS)_6_]	(BenzMIm)_3_[Cr(NCS)_6_]	(BMIm)[Cr(NCS)_4_(Pyr)_2_]	(EMIm)[Cr(NCS)_4_(Pic)_2_]
average Cr‒N	2.01	2.00	2.00	1.99/2.08 (Cr‒*L*)	1.99/2.08 (Cr‒*L*)
average N‒C	1.14	1.14	1.16	1.16	1.16
average C‒S	1.62	1.62	1.63	1.62	1.62
short. *D*‒H···*A*					
C‒H	0.93	0.93	0.99	0.95	0.95
H···S	2.72	2.99	2.86	2.77	2.95
C···S	3.620 (2)	3.682 (8)	3.570 (4)	3.647 (3)	3.661 (3)
C‒H···S [°]	164	133	129	155	133

## Data Availability

Crystallographic data can be obtained free of charge from the Cambridge Crystallographic Data Center: CCDC 1988743 for (DMIm)_3_[Cr(NCS)_6_], CCDC 1988745 for (EMIm)_3_[Cr(NCS)_6_], CCDC 1988741 for (BenzMIm)_3_[Cr(NCS)_6_], CCDC 1988742 for (BMIm)[Cr(NCS)_4_(Pyr)_2_], and CCDC 1988744 for (EMIm)[Cr(NCS)_4_(Pic)_2_].
